# 224 Longitudinal associations between flexibility, motor competence, and self-reported physical activity during adolescence

**DOI:** 10.1093/eurpub/ckae114.085

**Published:** 2024-09-26

**Authors:** Mikko Huhtiniemi, Timo Jaakkola

**Affiliations:** University of Jyväskylä, Jyväskylä, Finland; University of Jyväskylä, Jyväskylä, Finland

## Abstract

**Purpose:**

This study investigated the longitudinal associations between flexibility, motor competence, and self-reported physical activity among Finnish adolescents over four years. Previous research indicates a lack of longitudinal studies exploring the developmental patterns of flexibility, motor competence, and physical activity.

**Methods:**

At baseline, 1147 (582 boys, 565 girls) Finnish adolescents aged 11.27 (0.33) years participated in the study. Data was collected annually in five time points when students were in Grades 5 to 9. Students’ flexibility was measured using squat, lower back extension, and left and right shoulder stretch. Motor competence was measured using the throwing-catching combination, continuous 5-leaps, and lateral jumping tests. Physical activity was measured using a questionnaire. A Random-Intercept Cross Lagged Panel Model (RI-CLPM), including repeated measures (within-level) and latent levels (between-level) of flexibility, motor competence, and physical activity was used to analyze the data. Sex and body mass index (BMI) were used as covariates in the analysis.

**Results:**

At the between-level, a stable positive association over four years was found between flexibility and motor competence [.36, (.04)] and between motor competence and physical activity [.47, (.04)]. However, flexibility was not found to be associated with self-reported physical activity over time. The covariate results showed that being a girl was negatively linked to motor competence [-.12, (.03)] and physical activity [-.12, (.04)] but positively to flexibility [.41, (.03)]. Higher BMI was negatively linked to all study variables. The results at the within-level also revealed some cross-lagged relationships among the study variables.

**Conclusions:**

The findings of this study indicate that students’ flexibility is positively linked to motor competence during adolescence. In other words, students with higher flexibility also have higher motor competence, and vice versa. As flexibility and physical activity were not associated over time, it might be that the positive development of flexibility is more related to participating in activities with diverse motor skills demands rather than meeting the physical activity guidelines.

**Funding source:**

The Finnish Ministry of Education and Culture funded this study.

